# Antiseptics in Surgery

**Published:** 2010-05-27

**Authors:** Tobias Hirsch, Hans-Martin Seipp, Frank Jacobsen, Ole Goertz, Hans-Ulrich Steinau, Lars Steinstraesser

**Affiliations:** ^a^Department of Plastic Surgery, Ruhr-University Bochum, Bochum, Germany; ^b^Department of Environmental Engineering and Biotechnology, University of Applied Science Giessen-Friedberg, Giessen, Germany

## Abstract

**Background:** Wound healing is a complex process, with many potential factors that can delay or complicate healing. Bacterial infection is one of the most dangerous complications once the skin barrier is destroyed. The search for optimal treatment of chronic and infected wounds is an ongoing challenge for healthcare professionals. **Methods:** This article discusses recent findings in the field of wound antiseptics, its antibacterial efficacy, cell toxicity, and compatibility with wound dressings. **Results:** Skin antiseptics are daily used for wound cleansing to reduce the bacterial burden. However, there is little evidence concerning the antimicrobial efficacy, cytotoxicity of host cells, and compatibility with commonly used wound dressings. Recent findings show high toxicity and significant incompatibilities with wound dressings for some antiseptics. **Conclusion:** Antiseptics are widely used in hospitals worldwide to reduce, inactivate, or eliminate potentially pathogenic microorganisms. Current studies show that widely used wound antiseptics show relevant cytotoxicity and cross-reactivity with certain wound dressings. Future research should particularly focus on cytotoxicity, mechanisms of bacterial resistance toward skin antiseptics and wound irrigants, as well as compatibility and cross-reactivity with wound dressings.

Surgeons are constantly challenged to find the optimal treatment of difficult-to-heal wounds, such as chronic ulcers, trauma-induced wounds, and deep burns. Open wounds, particularly in diabetic and immunosuppressed patients, are susceptible to invading pathogens such as bacteria. Chronic wounds present an attractive environment for bacterial infection, and more than 80% of leg ulcers are colonized by bacteria.^1^,^2^ Bacterial colonization is associated with delayed wound healing and causes severe morbidity from sepsis and multiorgan failure.^3^,^4^ In the United States, delayed wound healing and bacterial infection due to diabetes are the leading causes of nontraumatic amputations (approximately 71,000 per year or 190 per day).^5^,^6^ At present, *Staphylococcus aureus* is the most common single isolate in chronic wounds (76% in foot ulcers) leading to impaired wound healing.^7^ Methicillin-resistant *S aureus* (MRSA) has become endemic in some hospitals,^8^ and in 2002, the first clinical isolate of vancomycin-resistant *S aureus* was identified in a diabetic patient with a foot ulcer.^7^,^9^

Primary strategies used to prevent and treat wound infection include systemic antibiotics and topical antiseptics/antibiotics. Insufficient accumulation in the soft tissue is still a major limitation of systemic antibiotics. Furthermore, systemic antibiotics struggle with increasing bacterial resistance and wound colonization with multiresistant strains.^3^,^8^ Thus, their clinical employment remains controversial.^10^

Topical antiseptics therefore play a key role for the treatment of wounds in current clinical practice. The philosophy behind local delivery of skin antiseptics is to raise tissue levels of antimicrobials to a level where sensitive and relatively insensitive organisms are inhibited and to avoid potential systemic side effects of high-dose antibiotics.

The first modern, chemically derived antiseptic agent was discovered by Friedlieb Ferdinand Runge in 1834, describing the structure and properties of carbolic acid (phenol).^11^ A further monumental advance toward the improvement of wound-healing outcome came from the work of Joseph Lister. The famous surgeon was the first to employ this striking agent in March 1865 in a complicated case of tibia fracture.^12^,^13^ In 1867, he described his technique for the use of carbolic acid spray for surgical antisepsis and direct prophylaxis of high-risk wounds.^12^,^13^ Within approximately 20 years, the aseptic techniques of Semmelweis and surgical antisepsis based on Lister's principles became the standard of care. In 1919, Alexander Fleming stated, “Antiseptics will only exercise a beneficial effect in a septic wound if they possess the property of stimulating or conserving the natural defensive mechanism of the body against infection.”^(p. 127)^ He further proclaimed that in estimating the value of an antiseptic, it is more important to study its effects on tissues than any effects on bacteria.^14^

Because antiseptics often have to be applied on human skin and wounds for therapy, it is important to evaluate their efficacy and the possible cytotoxicity. However, this important fact has been neglected in the past and still little is known about the cytotoxicity of clinically used skin antiseptics to date. Furthermore, compatibility of wound dressings with skin antiseptics is hardly investigated to date. This fact seems to be alarming since wound dressings present a huge market and these products are used widely by different healthcare professionals.

## ANTIBACTERIAL EFFICACY

In a recent study, we investigated antibacterial efficacy of 5 commonly used local antiseptics.^15^ Octenisept (octenidine), Lavasept, Prontosan (polyhexamethylene biguanide [PHMB]), Braunol, and Betaisodona (povidone-iodine) were tested for their antibacterial activity against 2 gram-positive (*S aureus* and *Enterococcus faecalis*) and 2 gram-negative *(Pseudomonas aeruginosa* and *Escherichia coli*) strains. Therefore, different dilutions (1%–20%) of the antiseptic solutions were investigated and minimal inhibitory concentration was determined. The analyzed bacterial strains were susceptible, in different degrees, to all the antibiotics tested, showing that all 5 antiseptics possess antibacterial effects. However, differences in their specific effectiveness were evident: Lavasept, Prontosan, and Octenisept show a strong antibacterial effect in every concentration tested (1%–20%), whereas povidone-iodine–based antiseptics require higher concentrations to completely inhibit bacterial growth (3%–7.5%; Table 1).

## ENHANCED RESISTANCE AGAINST LOCAL ANTISEPTICS IN MRSA STRAINS

Resistance of bacterial strains toward antibiotic agents is well described and well known in daily healthcare needs. Hirsch et al^16^ investigated antibacterial efficacy of PHMB-based wound antiseptics and wound irrigants toward susceptible *S aureus* strains and MRSA.

Regular American Type Culture Collection (ATCC)-listed *S aureus* strains were compared with MRSA strains (ATCC, Manassas, Va, and “International Basic Set,” Robert-Koch-Institute, Wernigerode, Germany) with the PHMB-based products Prontosan and Lavasept at a 0.005% concentration.

For each of the 4 MRSA strains, highly significant, elevated resistance toward the susceptible control strains was detected: MRSA strains showed an average 160-fold increased resistance, with a maximum of 400-fold. This study shows that antibiotic-resistant *S aureus* strains possess highly significant, elevated resistance toward local antiseptics compared with antibiotic-sensitive control strains.

## CYTOTOXICITY

**Figure 1:**
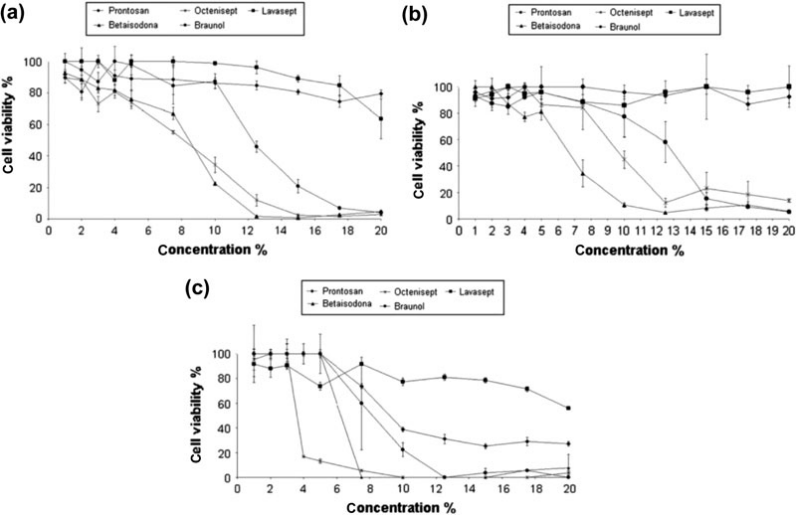
Cytotoxicity of antiseptics in skin cells. (*a*) In HaCaT cells, Lavasept and Prontosan show little toxicity. Betaisodona-treated cells show no vitality at a concentration of 12.5%, followed by Octenisept at 15% and Braunol at 20%. (*b*) Human keratinocytes are barely affected by Lavasept and Prontosan. Braunol and Octenisept show low cytotoxicity up to concentrations of 12.5%, followed by a marked decrease in cytotoxicity. Betaisodona is the most toxic agent. (*c*) In fibroblast, Lavasept showed the best result. Prontosan showed low toxicity. Braunol has low toxicity within low concentrations, followed by a linear decrease of 0% in cell viability. At 10%, Octenisept shows a decrease of 0%, followed by Betaisodona (0% cell viability at 7.5%).

To assess cytotoxic effects of local skin antiseptics at different concentrations, cell toxicity (MTT) and proliferation (BrDU) assays were performed in our study mentioned previously.^15^ Therefore, studies on primary human keratinocytes and fibroblasts, as well as HaCaT cell line, were performed (MTT assay and BrdU-ELISA) at 1% to 20% concentrations. In HaCaT cells, all skin antiseptics showed toxic effects toward the cells. However, Lavasept and Prontosan induced only moderate toxicity, whereas Betaisodona and Octenisept showed strong effects, followed by Braunol. These findings were confirmed in the primary human keratinocytes: Lavasept and Prontosan showed low toxicity, whereas Betaisodona, Octenisept, and Braunol had significant impact on cell viability (Fig 1).

To analyze the impact of skin antiseptics on proliferative activity of the cells, the BrDU-ELISA assay was performed. The data show that Lavasept and Prontosan had little to no effect on proliferative activity, whereas Betaisodona, Octenisept, and Braunol showed significant toxicity in all 3 cell types assessed (Betaisodona > Octenisept > Braunol). Generally, it turns out that fibroblasts are more susceptible to skin antiseptics than primary keratinocytes and the keratinocyte cell line HaCaT. Taken these data together, 3 of 5 skin antiseptics revealed significant alterations regarding cell viability and proliferation.

Langer and coworkers^17^ investigated the influence of local antiseptics on skin microcirculation in vivo and reported that all antiseptics assessed (70% ethanol, Softasept, Octenisept, and Lavasept) influenced skin microcirculation with regard to blood vessel leakage, functional capillary density, and red blood cell velocity when compared with the saline control. They reported that Octenisept had the least impact on microcirculatory parameters.^17^ Müller and Kramer^18^ investigated the antimicrobial effect of 12 different skin antiseptics, including PVP-iodine, octenidine, and PHMB solutions, toward *E coli* and *S aureus*. At the same time, cytotoxic effects in fibroblasts were analyzed. The authors concluded that octenidine and PHMB solutions were the most suitable agents assessed in their study.^18^ However, in an earlier study, the same authors stated that povidone-iodine seems to be the most tolerated antiseptic in comparison with chlorhexidine, octenidine, or PHMB solutions.^19^ Kalteis et al^20^ assessed the tissue compatibilities of Dibromol (sodium 3,5-dibromo-4-hydroxy benzenesulfonate), Kodan (propanol), Jodobac (povidone iodine), Octenisept, 0.2% Lavasept, hydrogen peroxide, 0.5% chlorhexidine digluconate, and 60% 2-propanol in the hen's egg test chorion-allantoic membrane (HET-CAM). The authors found that the most severe tissue toxicity being induced by 0.5% chlorhexidine digluconate and Kodan. Irritating values were determined for Dibromol, Octenisept, and 60% 2-propanol. Moderate vascular injuries were caused by Jodobac. Lavasept and hydrogen peroxide showed no tissue toxicity.^20^

## COMPATIBILITY

A primary strategy to prevent and treat infection in chronic wounds is the use of topical antiseptics, wound-irrigating agents, and wound dressings at the same time. Interestingly, the interaction of antiseptics with commonly used wound dressings is hardly investigated to date.

In a current study, we analyzed the antimicrobial activity of antiseptics and wound-irrigating agents with commercially available wound dressings: Five clinically used antiseptics and wound-irrigating agents (Prontosan, Lavasept, Braunol, Octenisept, and Betaisodona) were tested in the presence or absence of 42 wound dressings against *S aureus*. Antibacterial activity was determined by disc diffusion assay. In this study, povidone-iodine–based products showed sufficient antimicrobial activity in 64% to 78% of the combinations assessed (*P* > .01). The octenidine derivative Octenisept showed sufficient antimicrobial activity in 54% of combinations. PHMB derivatives demonstrated sufficient antimicrobial activity in 32% of the combinations. This study revealed that commonly used wound dressings dramatically reduce antibacterial activity of clinically used antiseptics and wound-irrigating agents in vitro.^21^

Other studies have shown that modern wound dressings show no significant improvement in wound healing. A Cochrane meta-analysis of the topical treatment of wounds with silver as the antimicrobial agent identified 3 randomized controlled clinical trials comprising a total of 847 participants. One trial compared silver-containing foam (Contreet) with hydrocellular foam (Allevyn) in patients with leg ulcers, the second trial compared a silver-containing alginate (Silvercel) with an alginate alone (Algosteril), and the third trial compared the silver-containing foam Contreet with best local practice in patients with chronic wounds. The data from these 3 trials demonstrated that silver-containing foam dressings did not significantly increase complete ulcer healing as compared with standard foam dressings or best local practice after up to 4 weeks of follow-up, although a greater reduction in ulcer size was observed with the silver-containing foam. The authors stated that there is insufficient evidence to recommend the use of silver-containing dressings for the treatment of infected or contaminated chronic wounds.^22^ In a meta-analysis by Nelson and Bradley,^23^ investigating dressings and topical agents for arterial leg ulcers to determine whether topical agents and wound dressings affect the rate of healing in arterial ulcers, only 1 trial met the inclusion criteria. This small trial compared ketanserin ointment with vehicle alone. The trial was too small and the follow-up period was too short to be able to determine whether there was any difference in healing rates.^23^ In another Cochrane review, the authors performed an extensive literature search in the field of dressings for healing venous leg ulcers. Forty-two randomized controlled studies were identified that met the inclusion criteria. The main dressing types that were evaluated were hydrocolloids (*n* = 23), foams (*n* = 6), alginates (*n* = 4), hydrogel dressings (*n* = 6), and a group of miscellaneous dressings (*n* = 3). There was no evidence that any particular dressing type was better than any others in terms of number of ulcers healed. At present, the evidence base does not suggest that hydrocolloids are more effective than simple low-adherent dressings used beneath compression. For other comparisons, there was insufficient evidence.

## CONCLUSION

Antiseptics are used in hospitals worldwide to reduce, inactivate, or eliminate potentially pathogenic microorganisms. Current studies show that widely used wound antiseptics show relevant cytotoxicity and cross-reactivity with wound dressings. Furthermore, antibiotic-resistant *S aureus* strains (MRSA) show highly significant, elevated resistance toward local antiseptics. Future research should particularly focus on cytotoxicity, mechanisms of bacterial resistance toward skin antiseptics, and wound irrigates, as well as compatibility and cross-reactivity with wound dressings. The science of antiseptic activity and efficacy testing is complex and further research would benefit patient care and prevent negative side effects.

## Figures and Tables

**Table 1:** Antibacterial testing of local antiseptic against gram-positive and gram-negative bacteria*

	Concentration, %											
	20	17.5	15	12.5	10	7.5	5	4	3	2	1	Control
Braunol												
*Staphylococcus aureus* ATCC 29213									+	+	+	+
*Enterococcus faecalis* ATCC 29212								+	+	+	+	+
*Escherichia coli* ATCC 25922								+	+	+	+	+
*Pseudomonas aeruginosa* ATCC 27853						+	+	+	+	+	+	+
Betaisodona												
*Staphylococcus aureus* ATCC 29213											+	+
*Enterococcus faecalis* ATCC 29212										+	+	+
*Escherichia coli* ATCC 25922										+	+	+
*Pseudomonas aeruginosa* ATCC 27853							+	+	+	+	+	+
Octenisept												
*Staphylococcus aureus* ATCC 29213												+
*Enterococcus faecalis* ATCC 29212												+
*Escherichia coli* ATCC 25922												+
*Pseudomonas aeruginosa* ATCC 27853												+
Lavasept												
*Staphylococcus aureus* ATCC 29213												+
*Enterococcus faecalis* ATCC 29212												+
*Escherichia coli* ATCC 25922												+
*Pseudomonas aeruginosa* ATCC 27853												+
Prontosan												
*Staphylococcus aureus* ATCC 29213												+
*Enterococcus faecalis* ATCC 29212												+
*Escherichia coli* ATCC 25922												+
*Pseudomonas aeruginosa* ATCC 27853												+

*Plus sign (+) indicates bacterial growth. Braunol showed antibacterial effect at a minimal concentration of 4% against *S aureus*. At a concentration of 5%, *E faecalis* and *E coli* were inhibited, followed by *P aeruginosa* at 10%. Betaisodona shows inhibitory effect against *S aureus* at 2% concentration. Antibacterial effect against *E faecalis* and *E coli* was detected at a concentration of 3%, whereas a concentration of 7.5% is necessary to obtain effectiveness against *P aeruginosa*. For Lavasept, Prontosan, and Octenisept, no bacterial growth could be detected.
